# Plasma proteins associated with chronic graft-versus-host disease organ involvement

**DOI:** 10.1172/jci.insight.209042

**Published:** 2026-07-28

**Authors:** Stephanie J. Lee, Corey Cutler, Ningxin Ma, Timothy W. Randolph, George L. Chen, Joseph Pidala, Betty K. Hamilton, Carrie L. Kitko, Sally Arai, Najla El Jurdi, Lynn Onstad, Motoko Koyama, Catherine J. Lee, Sophie Paczesny, Geoffrey R. Hill

**Affiliations:** 1 Fred Hutchinson Cancer Center, Seattle, Washington, USA.; 2 Division of Transplantation and Cellular Therapy, Dana-Farber Cancer Institute, Boston, Massachusetts, USA.; 3 The University of Texas MD Anderson Cancer Center, Houston, Texas, USA.; 4 Department of Blood and Marrow Transplantation and Cellular Immunotherapy, Moffitt Cancer Center, Tampa, Florida, USA.; 5 Taussig Cancer Institute, Cleveland Clinic, Cleveland, Ohio, USA.; 6 Vanderbilt University Medical Center, Nashville, Tennessee, USA.; 7 Stanford Blood and Marrow Transplantation and Cellular Therapy Division, Stanford School of Medicine, Stanford University, Stanford, California, USA.; 8 Immune Deficiency Cellular Therapy Program, Center for Cancer Research, National Cancer Institute, NIH, Bethesda, Maryland, USA.; 9 Hollings Cancer Center and Department of Pharmacology and Immunology, Medical University of South Carolina, Charleston, South Carolina, USA.ZZZZÆ

**Keywords:** Clinical Research, Hematology, Oncology, Biomarkers, Bone marrow transplantation, Cytokines

## Abstract

BACKGROUND. Previous studies identified plasma proteins associated with chronic graft-versus-host disease (cGVHD). The goal of this cross-sectional study was to evaluate whether plasma biomarkers were associated with specific organ manifestations to help guide treatment choice.

METHODS. Plasma proteins were measured in patients with cGVHD (*N* = 695) from Chronic GVHD Consortium studies. Correlations of plasma protein levels with individual organ involvement were tested with a *P* value of 0.05 or less considered significant after Benjamini-Hochberg adjustment and adjustment for 5 baseline clinical variables.

RESULTS. Median time from cGVHD diagnosis to blood draw was 0.9 months (IQR 0.1–9.5). Donors were 50% HLA-matched unrelated, 32% matched related, and the remainder were umbilical cord blood, haploidentical, or mismatched unrelated donors. Methotrexate and calcineurin inhibitor prophylaxis for acute GVHD was used in 53% of patients. Overall, 326 (47%) had moderate and 244 (35%) had severe cGVHD with the following organ involvement at time of blood draw: skin (67%), mouth (60%), eye (49%), joint (34%), GI (31%), lung (23%), and liver (17%). After adjustment for batch effects and patient and transplant characteristics, 14 plasma proteins were associated with organ involvement with independent AUCs of 0.7–0.8. All organs except the eye were associated with at least 1 biomarker. However, no combination of plasma proteins improved model fit after adjusting for patient and transplant clinical variables.

CONCLUSION. Correlations between plasma proteins and organ involvement were identified but are not actionable. Our future investigations will focus on more granular and immediately proximal determinants of cGVHD biology in both blood and tissue.

## Abstract 2

Largest study of chronic GVHD plasma proteins

## Introduction

Chronic graft-versus-host disease (cGVHD) is a major cause of long-term morbidity and mortality in allogeneic hematopoietic cell transplantation (HCT) recipients. cGVHD commonly involves the skin, mouth, and eye, but may also affect the liver, lung, fascia, genital tract, kidney, muscle, and the hematopoietic and nervous systems. Newer GVHD-prophylaxis approaches are associated with lower rates of cGVHD than older regimens, but between 10% and 30% of people still develop cGVHD that is severe enough to require systemic immunosuppression (1). Treatment is often prolonged, with approximately 15% of treated patients still on immunosuppression 10 years later (2). cGVHD is a heterogeneous disease and patients display different combinations of organ involvement.

The pathobiology of cGVHD has been summarized as initial cytokine-based inflammation followed by dysregulated immunity leading to pathologic tissue repair (3, 4). Multiple plasma biomarkers have been reported to be associated with cGVHD, including elevated levels of IL-1β (5), sIL-2-Ra (6, 7), IL-6 (8), IL-8 (8), IL-15 (9), IL-17A/F (8), TNF-α (5, 8, 10, 11), TGF-β (12–14), chemokine (C-X-C motif) ligand 9 (C-X-C motif chemokine ligand 9 [CXCL9], also known as MIG) (15, 16), CXCL10 (IP-10) (17), CCL15 (18), sBAFF (19, 20), and APRIL (21) and decreased IL-10 (5, 7, 10, 22) and IL-2 (23). Others such as IL-4 are controversial (23–25). In a study with 2 replication cohorts, soluble IL-1 receptor-like 1 (IL1RL1, previously stimulation 2 [ST2]), CXCL9, matrix metalloproteinase 3 (MMP3), and osteopontin (OPN) were increased in patients with cGVHD compared with patients without cGVHD (15). Soluble CD163 is a macrophage scavenger receptor, and elevated levels at day +80 were associated with de novo cGVHD (26). Dickkopf-related protein 3 (DKK3), discovered in patients with sclerotic skin and lung involvement, has been associated with overall cGVHD. In general, these studies compared fewer than 200 patients with cGVHD to control patients without cGVHD. They did not consider or did not find correlation with individual organ involvement (Tables 1 and 2).

We sought to identify plasma proteins that correlate with specific organ involvement in a large cohort of adult patients with well-characterized cGVHD. We hypothesized that plasma protein analysis might reflect tissue-specific pathogenesis and help explain heterogeneous organ involvement. Organ-specific treatment approaches could then be tailored if aberrant pathways were identified.

## Results

### Patient characteristics.

Patient characteristics are shown in Supplemental Table 1; supplemental material available online with this article; https://doi.org/10.1172/jci.insight.209042DS1 Sixteen institutions enrolled patients between 2007 and 2019, with over half accrued at Fred Hutchinson Cancer Center. The population was predominantly White (88%) and male (62%) who received peripheral blood grafts (90%). Acute myeloid leukemia, acute lymphoblastic leukemia, and myelodysplastic syndrome accounted for 63% of diagnoses. Approximately half the population received grafts from matched unrelated donors (50%), received calcineurin inhibitor and methotrexate acute GVHD (aGVHD) prophylaxis (53%), and had myeloablative conditioning (46%). Only 5 (1%) had haploidentical donors and 9 (1%) received posttransplant cyclophosphamide. Tables 3 and 4, and Supplemental Table 2 show GVHD characteristics at time of sample draw. cGVHD was mild in 18%, moderate in 47%, and severe in 35%; 63% were diagnosed within the previous 3 months. The most commonly involved organs were skin (67%), mouth (60%), and eye (49%). Prior grade II–IV aGVHD occurred in 48%. At the time of the blood sample, 60% were receiving prednisone and 48% were on a calcineurin inhibitor; 9.1% were not on any immunosuppression. Although most were continuing previous immunosuppressive agents, 73.1% had not started any new cGVHD-specific treatment right before the sample was collected. Previous treatments for cGVHD included calcineurin inhibitors (12.7%) and prednisone (10.2%). Less than 10% had previously received sirolimus, an antimetabolite (mycophenolate mofetil or methotrexate), extracorporeal photopheresis, or a B cell–targeting agent (BTK inhibitor or anti-CD20 monoclonal antibody) for cGVHD prior to sample acquisition. Three patients were on ruxolitinib, 1 on belumosudil, and none on axatilimab at the time of sample acquisition.

### Plasma proteins.

Supplemental Figure 1 shows the correlations of the plasma proteins with each other. Strong correlations were seen between CXCL9 and CXCL10, as expected. Other correlations (Pearson’s correlation > 0.5) could reflect (a) Th1-Th17 and myeloid activation with IL-12, IL-21, and GM-CSF, and (b) antigen-presenting cell and Th1 and B cell activation with IL-12, CXCL9/CXCL10, and BAFF.

### Association of individual plasma proteins with individual organ involvement, without adjusting for clinical variables.

**Figure 1:**
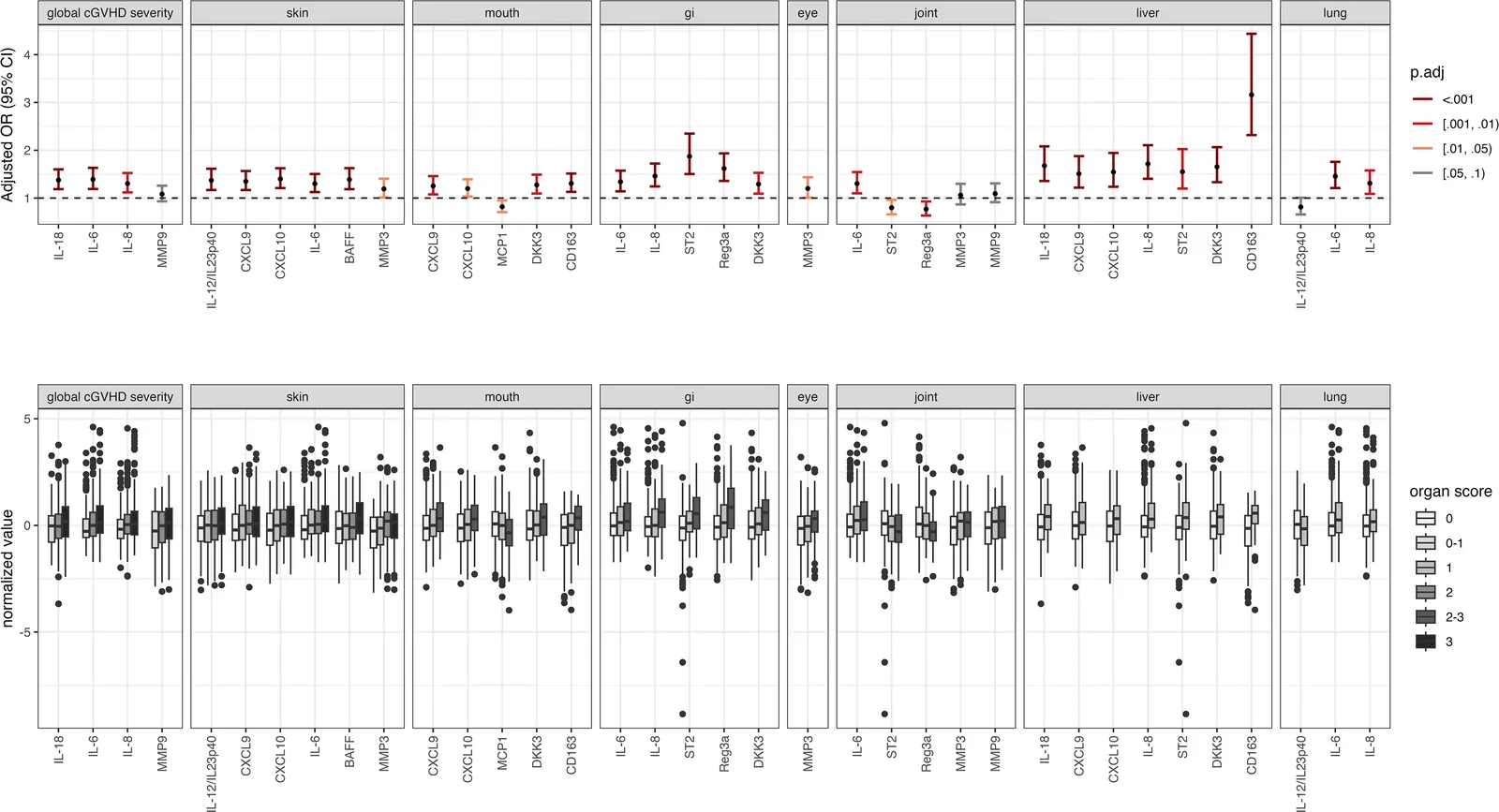
Plasma protein levels associated with various levels of organ involvement, not adjusting for clinical covariates.

Supplemental Table 3 shows the associations of the 19 plasma proteins with cGVHD organ involvement, adjusted for multiple testing but unadjusted for the 5 clinical variables. Five plasma proteins (GM-CSF, IL-12, IL-21, M-CSF, and IL-17A) were not associated with any organ involvement (at *P* < 0.05) and were not considered further. All organs except the eye had at least one plasma protein association (at *P* < 0.05). Despite the strong statistical associations, Figure 1 shows the overlapping distributions of plasma protein levels between different organ severity levels, presented as box-and-whisker plots and odds ratios (ORs).

### Association of individual plasma proteins with individual organ involvement, adjusting for clinical variables.

Table 5 shows the marginal association of the 14 remaining plasma proteins with cGVHD organ manifestations, adjusting for the following 5 clinical variables: patient age, patient sex, time between HCT and blood draw, time since cGVHD diagnosis (more or less than 3 months), and steroid dose, as a continuous variable. *P* values were adjusted for multiple testing. Thirteen plasma proteins: IL-18, CXCL9, CXCL10, IL-6, IL-8, MCP1, BAFF, IL1RL1 (ST2), REG3a, MMP3, DKK3, and CD163 remained significantly associated with at least one organ manifestation, and there were 30 associations among the 7 organ systems (Supplemental Table 3). Only 2 of these associations were previously reported in the literature: IL-6 and joint involvement (6) and REG3a and gastrointestinal (GI) involvement (27), while the others have not been previously reported. MMP9 was not statistically associated with any organ manifestations once models were adjusted for the 5 clinical variables, although MMP9 was associated with joint and liver in our univariable analyses. Some previous publications have used an OR of greater than 1.3 or less than 0.7 to declare clinical importance as well as statistical significance; if this criterion is applied, MCP1 and MMP3 would no longer be associated with any organs. If a higher OR greater than 1.5 is required for clinical importance, only 4 plasma proteins associated with liver involvement (IL-18, IL-8, IL1RL1 [ST2], and CD163) and 2 associated with GI involvement (IL1RL1 [ST2] and REG3a) would be considered clinically important.

Supplemental Figure 2A shows the area under the receiver operating characteristics curve (AUC) for joint involvement, as an example of high AUCs of 0.78 (0.71–0.85) for the 5 clinical variables alone without improvement after adding the plasma protein values. AUCs ranged from 0.77–0.81 using the different models for joint involvement. A similar pattern was seen for the other organs (data not shown). For the logistic regression model including only plasma proteins, the positive predictive value (PPV) was 0.76 (0.69–0.84) and the negative predictive value (NPV) was 0.46 (0.37–0.57) at the optimal cutoff. Thus, despite the high and usually acceptable AUCs for the correlative models, the PPV and NPV of the plasma proteins were modest. Supplemental Figure 2B shows the kernel-association testing procedure (KRV) coefficients demonstrating the very low explanatory value of plasma protein profiles for organ involvement.

Supplemental Table 4 displays the variables selected most often in our repeated application of the elastic net regression using the randomly chosen 80% training/20% sampling procedure for the 7 organs, as illustrated for joint in Supplemental Figure 2. In general, each of the 5 clinical variables was important in different models, with time since diagnosis important in 6 out of 7 models, years after transplant and patient age in 4 out of 7 models, and sex and prednisone dose in 3 out of 7 models.

### Clustering of plasma proteins and organs.

**Figure 2:**
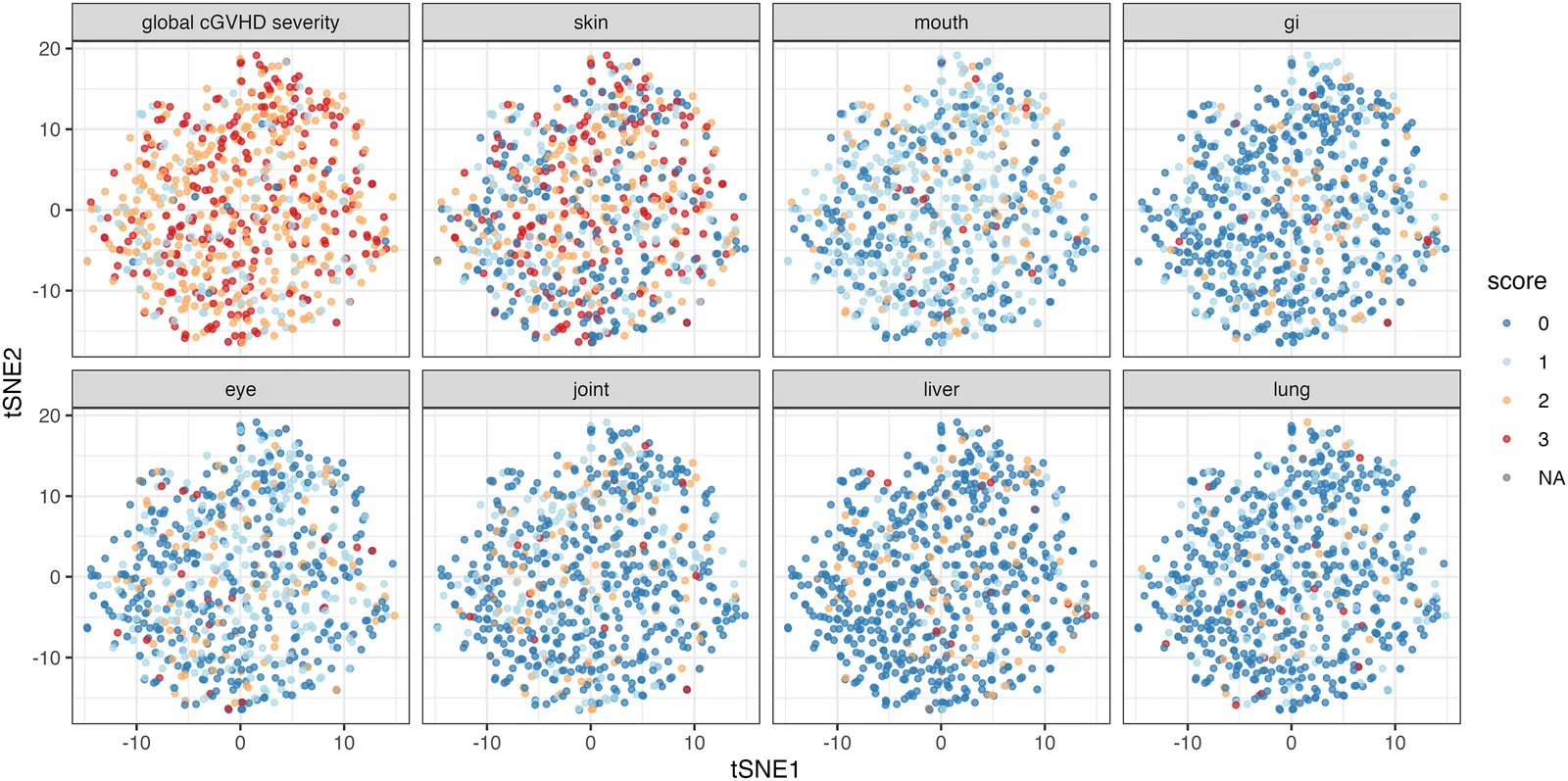
Dimensionality reduction using t-distributed stochastic neighbor embedding (tSNE) clustering of plasma proteins for each organ. The lack of identifiable clusters shows the lack of association between plasma protein profiles and organ involvement.

Figure 2 shows the results of dimensionality reduction using t-distributed stochastic neighbor embedding (tSNE) clustering of plasma proteins for each organ. We were unable to identify any meaningful clusters defined by plasma proteins using this method. The results using uniform manifold approximation and projection (UMAP) were similarly unrevealing (not shown).

## Discussion

In the largest study of plasma proteins in people with cGVHD, we found highly statistically significant correlations between several plasma proteins and individual organ manifestations, but plasma proteins did not improve model performance once patient age, sex, time from HCT to sample draw, time from cGVHD diagnosis to sample draw, and prednisone dose were included. In addition, we could not replicate findings previously reported in the literature, such as an association of joint involvement with MCP1 or BAFF, liver with BAFF, and lung with MMP3 or MMP9 (6, 28, 29). One possible explanation for this discrepancy is that we compared cGVHD patients with and without specific organ involvement, whereas other studies compared patients with and without cGVHD. If pathogenic mechanisms of cGVHD are shared over various target organs, our approach may have not detected associations.

The heterogeneous organ distribution of cGVHD has always been puzzling to clinicians. Organ manifestations that seem to have common clinical pathobiology such as fibrosis causing skin sclerosis and bronchiolitis obliterans syndrome (BOS) are not epidemiologically linked (30). Our study was designed to seek plasma proteins associated with specific organ involvement, hoping to identify targetable subgroups, although recent studies have suggested that this may be challenging (31). Indeed, some of the strongest associations we identified were markers of tissue damage and repair rather than upstream markers of inciting immunological events. Two of these markers, IL1RL1 (ST2) and REG3a, have been correlated with aGVHD, which approximately 50% of our cohort had experienced.

Many publications report statistically significant associations with AUCs of 0.7–0.8 between biomarkers and the general syndrome of cGVHD. Although we were successful in identifying diagnostic biomarkers, they did not improve the model AUCs once we adjusted for transplant and patient characteristics. One of our other goals was to identify pathway biomarkers that could help guide therapeutic choice for specific organ manifestations. Despite statistical significance in our study, overlap of protein values is substantial between those with and without organ involvement, causing the PPVs and NPVs to be disappointing. The highest values were achieved with joint involvement where a PPV of 76% and NPV of 46% means that using this biomarker model for joint-directed therapy would include 24% with false positive values who do not have joint involvement and exclude 54% with false negative values who do.

Another goal of studying biomarkers is to identify predictive biomarkers for responsiveness to different treatments or clinical outcomes such as time to next treatment or survival. These analyses are ongoing.

A number of study limitations are present. This is an adult population accrued over 2 decades. The transplant technologies represented do not reflect the most modern donor sources (haploidentical and mismatched unrelated donors) and GVHD prophylaxis regimens (posttransplant cyclophosphamide, novel T cell manipulation strategies, abatacept or ruxolitinib). The population is also heterogeneous in terms of transplant characteristics, time since cGVHD diagnosis, and prior lines of therapy, and 91% were on immunosuppression when the sample was drawn. Organ scoring using NIH criteria does not clearly distinguish fibrotic from inflammatory manifestations or active from fixed deficits. We did not apply our findings to an independent validation cohort, instead relying on bootstrapping for our prediction models. We did not include patients without cGVHD because we were interested in diagnostic biomarkers for individual organ involvement. Some of the patients in our cohort were included in reports by others (up to 198 patients in one study) and contributed to published associations (15–17, 26–29, 32–34). Finally, the cGVHD organ scoring system combines inflammatory and fibrotic cGVHD into 1 scale, although biomarkers may be more associated with one or the other phenotype. Future work will look at cleaner patient subsets with better-defined pathology, such as those with sclerosis, and also correlate plasma protein patterns with therapeutic response and longer-term outcomes.

We also experienced technical challenges. Several of the plasma biomarkers were below the limit of detection in our assay or otherwise failed quality control so could not be included. Samples were processed at collecting sites under similar processing protocols but likely varied in their time from draw to processing. Most patients were on immunosuppressive therapy at the time of sample collection, which may have altered the plasma profile. We controlled for corticosteroid dose but not other immunosuppressive agents. Because of the number of samples, most assays were tested in batches and we observed drift in assay controls over time. While these issues plague all biomarker studies, when combined with the heterogeneous population, detection of subtle associations was going to be particularly challenging, although this also reflects the reality of how such assays might be applied clinically.

Our results suggest that the measurement of soluble proteins in blood in patients with active cGVHD is unlikely to be a useful strategy to identify correlation with specific organ involvement. This may reflect limitations in assay sensitivity and/or tissue-restricted changes rather than systemic biology because soluble plasma proteins may have limited correlation with aberrant tissue-specific cGVHD pathophysiology. Cellular populations in blood and tissue may represent more stringent biomarkers and these studies are ongoing. Using scRNA-seq, we have identified aberrant pathways affecting Th17 and CSF-1 signaling in immune effector cells, potentially providing biomarkers of pathways more immediately proximal to cGVHD phenotypes (35). These studies provide better characterization of immune mediators and may identify targets for personalized cGVHD treatments. Host target tissue reactions may vary substantially and determine whether alloimmunity will be clinically apparent. The concept of tissue resilience has been proposed as an explanation for the variability in aGVHD (36) and may be operative in cGVHD. Studying tissues themselves, although challenging to obtain, may reveal more informative clues to pathobiology within individual patients (37–39).

In summary, although we found statistically significant associations between plasma proteins and organ involvement in cGVHD, we did not find actionable correlations. Future studies aiming to identify biomarkers should likely focus on immediately proximal cellular and molecular determinants of cGVHD biology in both blood and tissue.

## Methods

### Sex as a biological variable

Males and females were included. The study was not specifically powered to detect differences in biomarker performance based on sex and analyses were not adjusted unless otherwise specified.

### Patients

Male and female patients (*N* = 695) were included from 3 prospective, multicenter cohort studies run by the Chronic GVHD Consortium. Concurrent aGVHD manifestations were allowed as long as patients met the diagnostic criteria for cGVHD. Patients were excluded if they had evidence of persistent or progressive malignancy at the time of enrollment. Sex, race, and ethnicity were self-reported at enrollment using categories provided by study investigators and are summarized in Supplemental Table 1. Studies are registered at https://clinicaltrials.gov/

Study 1 (*n* = 146, NCT00637689) accrued patients between 2007 and 2012 to test the 2005 NIH cGVHD consensus criteria. Eligible patients were HCT recipients 2 years of age or older with cGVHD diagnosed according to the NIH consensus criteria who required systemic immunosuppressive therapy (40). Study 2 (*n* = 201, NCT01206309) enrolled adults between 2011 and 2014 to understand the natural history of cGVHD development. Blood samples were collected when patients developed cGVHD (30). Study 3 (*n* = 348, NCT01902576) enrolled patients between 2013 and 2019 to test NIH cGVHD response criteria. Participants were 7 years of age or older who had a diagnosis of cGVHD and were starting a new systemic immunosuppressive treatment (41).

Participants were selected from the 3 studies if they had an available frozen plasma aliquot with concurrent cGVHD organ scoring per NIH criteria. Patients were included irrespective of their prior and current immunosuppressive therapy exposure and time since cGVHD diagnosis.

We considered limiting the cohort to incident cases but decided to include both incident and prevalent cases for the following reasons: (a) we are interested in the full spectrum of organ manifestations, not just those seen in early presentations of cGVHD; (b) almost all patients are on immunosuppression at the time of blood sampling, either residual prophylaxis or treatment for aGVHD, so limiting to the incident cases would not yield a “pure” untreated cGVHD cohort; (c) one of our goals was to identify biomarkers to help guide treatment choice, but initial treatment for cGVHD is fairly established; and (d) prevalent cases comprise about 37% of the cases.

### Plasma assays

Heparin samples had been processed as soon as possible into peripheral blood mononuclear cells and plasma aliquots using standard methods. Samples were frozen at each site, then batch shipped to the biorepository. Tables 1 and 2 summarize the characteristics of the plasma proteins selected for testing. Plasma concentrations of IL-1β, IL-2, IL-4, IL-6, IL-8, IL-10, IL-12/IL-23p40, IL-13, IL-17A, TNF-α, and MCP1 were determined using the Cytometric Bead Array (CBA) system (BD Biosciences) according to the manufacturer’s protocol. In brief, samples and cytokine standards were incubated with mixed capture beads for 1 hour and then phycoerythrin-conjugated (PE-conjugated) streptavidin was added for 2 hours, followed by washing the beads. Samples were acquired with a FACSymphony A3 (BD Biosciences) and analysis was performed with FCAP Array Software v3.0 (BD Biosciences). Plasma concentrations of BAFF, IL-18, REG3a, GM-CSF, IFN-α, IFN-γ, IL-12p70, IL-21, IP10 (CXCL10), M-CSF, and MIG (CXCL9) were measured by Luminex multiplex assay according to the manufacturer’s protocol. In brief, samples and cytokine standards were incubated overnight with Luminex microbeads (1 unique bead population per cytokine) coated with cytokine-specific antibodies. Beads were washed and then incubated 1 hour with biotinylated anti-cytokine antibodies, washed again, and then incubated 30 minutes with the PE-streptavidin conjugate. After a final wash, the samples were acquired with a Luminex FLEXMAP 3D Instrument and analyzed with xPonent 4.2 software (Luminex). The sources and identifiers for reagents are listed in Supplemental Table 5.

Levels of IL-17A, IL1RL1 (ST2), CD163, DKK3, MMP3, and MMP9 were measured by enzyme-linked immunoabsorbent assays (ELISA). Proteins were measured using commercially available ELISA kits following the manufacturer’s recommendations and using a sequential ELISA approach previously described (15, 42). All samples and standards were tested in duplicate. All washes were performed using the Aquamax 2000 plate washer (Molecular Devices). Absorbance was measured immediately after termination of the substrate reaction using a SpectraMax ABS Plus plate reader and results were calculated using SoftMax Pro Version 7.1 (Molecular Devices). Antibody pairs, plasma dilutions, and ELISA parameters are described in Supplemental Table 5.

A total of 28 plasma proteins were measured. Note that IL-17A was measured both by CBA and ELISA. After exclusion of 7 proteins due to undetectable levels (IL-1β, IL-2, IL-4, IL-10, IL-13, IL-17A [by CBA], and TNF-α) and 2 (IFN-α and IFN-γ) due to uniformly high levels in normal controls, 19 were included in this analysis (IL-12p70, IL-12/IL-23p40, IL-18, IL-17A [by ELISA], IL-21, CXCL9, CXCL10, GM-CSF, M-CSF, IL-6, IL-8, MCP-1, BAFF, IL1RL1 [ST2], REG3a, MMP3, MMP9, DKK3, and CD163).

### Statistics

#### Plasma protein measurement processing.

For proteins analyzed by bead-based multiplex immunoassays, samples were processed in 15 batches, each containing those from 45–48 patients. To mitigate batch effects, plasma protein concentrations were log-transformed, then median-centered and scaled within each batch. Although the intent was to randomize the samples between runs, batches 1–4 and 12–15 were tested in the order they were pulled from storage. For batches 5–11 (47% of samples), samples from the 3 originating studies were randomized to ensure equal distribution on each plate. Downstream analyses were performed on the log-transformed batch-standardized data. ELISAs were run in 1 batch. Statistical analyses were conducted in R (version 4.4.1). Box indicates the interquartile range (25th–75th percentiles), center line indicates the median, and whiskers extend to the minimum and maximum values.

#### Clinical characteristics.

For each patient, we analyzed 7 organ-specific NIH scores (0 to 3) for skin, mouth, eye, joint/fascia, liver, GI tract, and lung, and an overall cGVHD severity score (mild, moderate, severe). Based on the proportions, liver and lung were dichotomized into present versus absent, whereas skin, mouth, GI, eye, joint, and overall severity were ordinal with 3 or 4 levels for maximum power. For cGVHD global severity, mild and unscored were combined. For example, global severity cannot be calculated for patients with only an asymptomatic mouth cGVHD score of 0 or other non-scored manifestations. For mouth, eye, GI, and joint, scores 2 and 3 were combined due to low numbers. Patients with missing organ scoring were excluded from individual analyses. Information about genital tract involvement was missing for the majority of patients and was excluded entirely from consideration. Based on previous studies, we adjusted for the 5 following clinical variables: age at sample collection, sex, time from transplant to sample collection, time from cGVHD diagnosis to sample (within 3 months, 3 or more months), and steroid dose (mg/day) at time of sample. Current treatments at the time of sample acquisition were defined as “concurrent immunosuppressive agents,” while immunosuppressive agents given since the diagnosis of cGVHD but not taken at the time of sample draw were called “past cGVHD treatments.” Due to the complexity of the analysis, we did not adjust for prior or concurrent immunosuppressive agents other than steroid dose.

#### Regression analyses.

Univariate logistic regression and ordinal logistic regression were used to test the marginal associations between 19 individual plasma proteins and 7 organ scores plus the cGVHD global scores. *P* values from the regression analyses were adjusted for multiple testing using the Benjamini-Hochberg procedure (false discovery rate, FDR). Plasma protein-organ score associations that remained significant after multiple-comparison adjustment were further evaluated using regression models adjusting for the 5 clinical covariates.

#### Correlation analyses.

Organ involvement and overall severity scores were dichotomized (present vs. absent) in prediction models to correlate plasma protein levels with organ manifestations. Three sets of variables were considered: (a) clinical covariates alone, (b) plasma proteins alone, and (c) clinical covariates and plasma proteins combined. Models evaluated included logistic regression, elastic net (43), random forest (original, balanced, and weighted variants), XGBoost (original, weighted, and DART) (44), and Bayesian Additive Regression Tree (BART) (45). Data were randomly partitioned into a training set (80% of the samples) to estimate model parameters, and a test set (20%) to evaluate predictive performance. Model performance was assessed on the test sets using the AUC, PPV, and NPV. To ensure robust estimates of performance, the entire process was repeated 100 times with different random splits of the training/test sets. The final performance metrics were reported as averages over the 100 test sets with empirical 95% CIs.

With the goal of assessing which variables are most related to organ involvement, we implemented an elastic-net generalized linear regression model (46) using the 5 clinical variables and all protein measures as predictors of binary symptom scores as outcome. This penalized regression procedure sparsely selects the most strongly associated variables the L1 (“lasso”) penalty term while accounting for correlation among the variables by including an L2 (“ridge”) penalty term. The trade-off between these 2 regularization terms was set to α = 0.95, in order to promote sparsity with a larger L1 penalty.

#### Proteomic profile “global” associations.

In an evaluation of the proteomic-wide association of the remaining 18 proteins (IL-17A was removed due to its high proportion of missingness) with organ scores, a kernel-association testing procedure, or “KRV” test (47), was applied to each of the 7 organ scores. This test provides a single *P* value for the overall correlation between a set of multivariate measurements collected on the same individuals. An overall *R*^2^ is also reported, i.e., the proportion of variance in the organ scores that is predicted by the proteomic profiles.

### Study approval

All sites obtained IRB approval, and all participants provided written informed consent before enrollment in accordance with the principles of the Declaration of Helsinki.

### Use of artificial intelligence

ChatGPT 5.2 was used to draft Tables 1 and 2. All table entries were confirmed by the authors through review of listed references and edited before submission.

### Data availability

Deidentified participant-level data and analytic code supporting the findings of this study are available from the corresponding author upon reasonable request, subject to institutional policies governing participant confidentiality. The numerical values underlying all graphical data are provided in the Supporting Data Values file.

## Author contributions

Designed research: SJL, TWR, SP, and GRH. Collected data: CC, GLC, JP, BKH, CLK, SA, and NEJ. Analyzed data: NM, TWR, and LO. Drafted the manuscript: SJL. Approved final submission: SJL, CC, NM, TWR, GLC, JP, BKH, CLK, SA, NEJ, LO, MK, CJL, SP, and GRH.

## Conflict of interest

SJL has received consulting fees from Novartis, Incyte, Sanofi, Orca Bio, Sonoma, and GSK; research funding from AstraZeneca, Incyte, Kadmon, Pfizer, Sanofi, and Syndax; and study medications provided by Janssen. SJL is on clinical trial steering committees for Incyte and Sanofi and is on the Board of Directors of the National Marrow Donor Program (uncompensated). CC has received consulting fees from Sanofi, Incyte, GSK, Graviton, Arog, OrcaBio, and CareDx. GLC received consulting fees from Sanofi. JP has received consulting fees from Incyte, Sanofi, and Deciphera and research funding from BMS, CTI Biopharma, Incyte, Sanofi, Johnson & Johnson, and AstraZeneca. BKH received consulting fees from Incyte and Sanofi; research funding from Incyte; and served on an adjudication committee for CSL Behring. CLK has served on the advisory boards for Incyte, Mesoblast, and Sanofi and has served on the GVHD adjudication committee for a clinical trial performed by CSL Behring. CJL has received consulting fees from Incyte, Sanofi, and Jazz and research funding from Incyte. CJL is on a clinical trial steering committee for Incyte. SP holds a patent on “Biomarkers and assays to detect chronic graft versus host disease” (US Patent 10,571,478 B2) that has been licensed to Eurofins/Viracor. GRH has consulted for Generon Corporation, NapaJen Pharma, iTeos Therapeutics, Commonwealth Serum Laboratories, Cynata Therapeutics, Neoleukin Therapeutics, and Incyte Pharma and has received research funding from Compass Therapeutics, Syndax Pharmaceuticals, Applied Molecular Transport, Serplus Technology, Heat Biologics, Laevoroc Oncology, iTeos Therapeutics, Genentech, Incyte Pharma, and Commonwealth Serum Laboratories.

## Funding support

This work is the result of NIH funding, in whole or in part, and is subject to the NIH Public Access Policy. Through acceptance of this federal funding, the NIH has been given a right to make the work publicly available in PubMed Central.

- NIH grants CA118953 (to SJL), CA163438 (to SJL), CA264921 (to SP), and CA168814 (to SP).
- NIH Intramural Research Program.

## Supplementary Material

Supplemental data

Supporting data values

## Figures and Tables

**Table 4:**
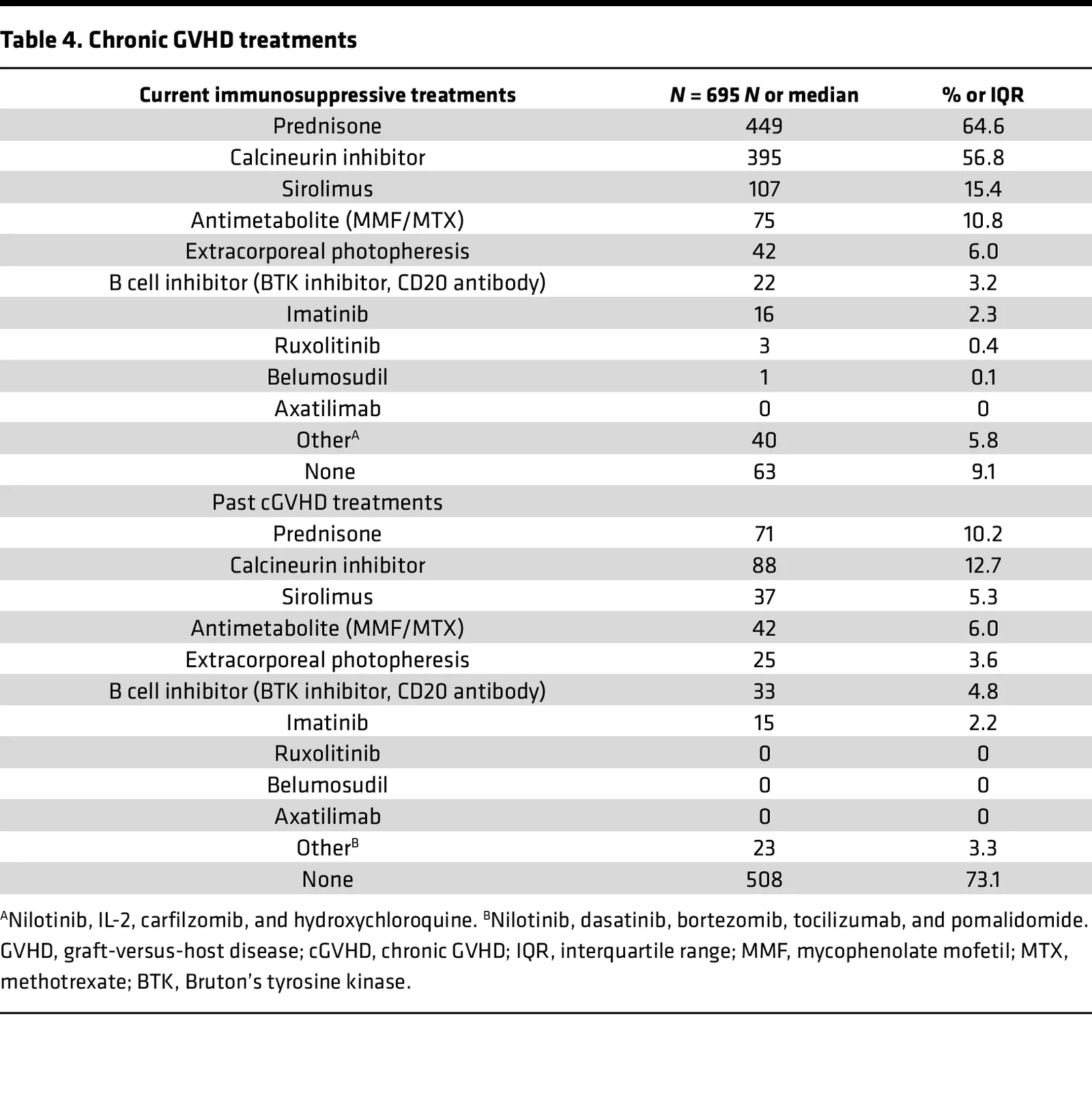
Chronic GVHD treatments

**Table 2:**
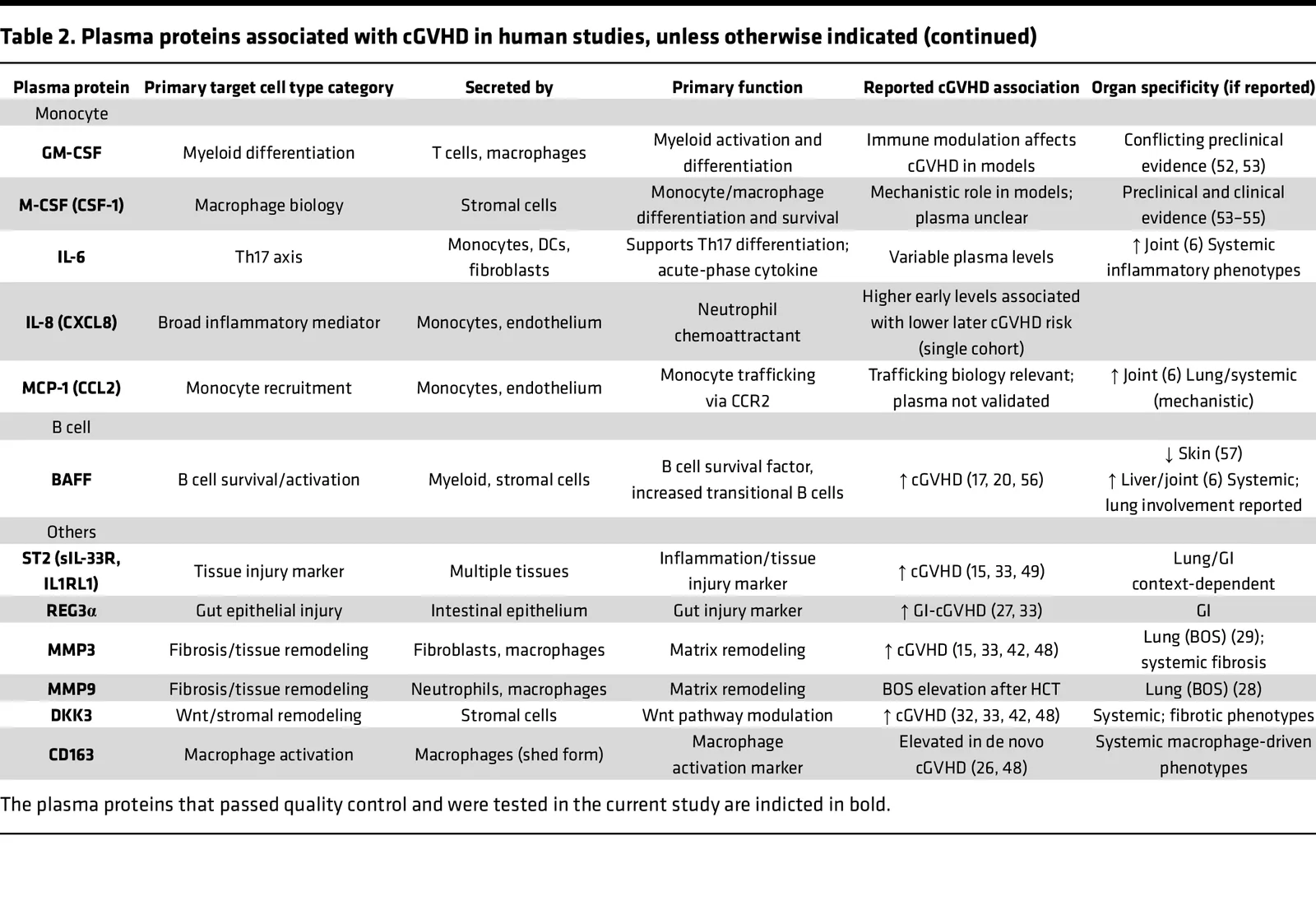
Plasma proteins associated with cGVHD in human studies, unless otherwise indicated (continued)

**Table 1:**
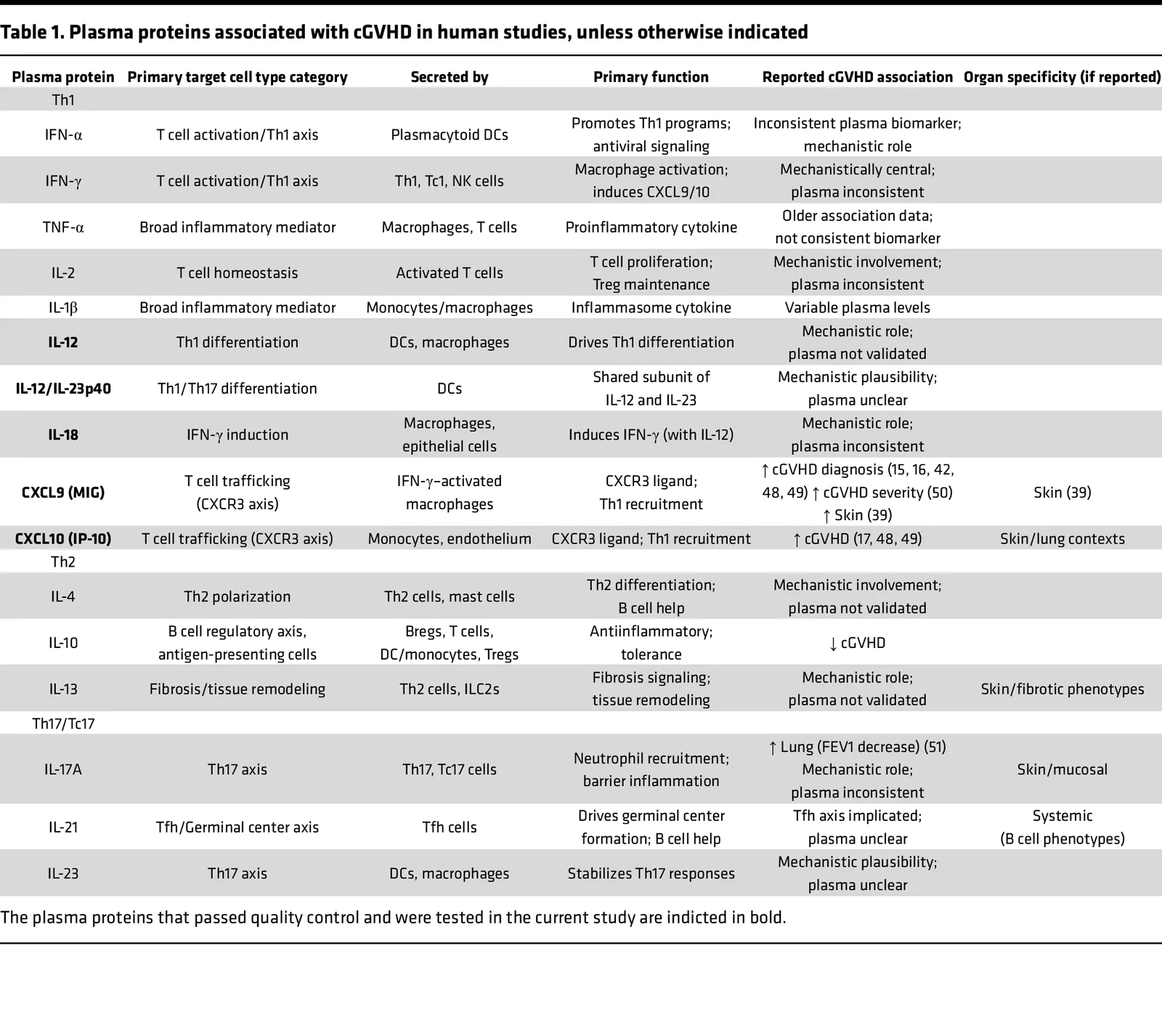
Plasma proteins associated with cGVHD in human studies, unless otherwise indicated

**Table 3:**
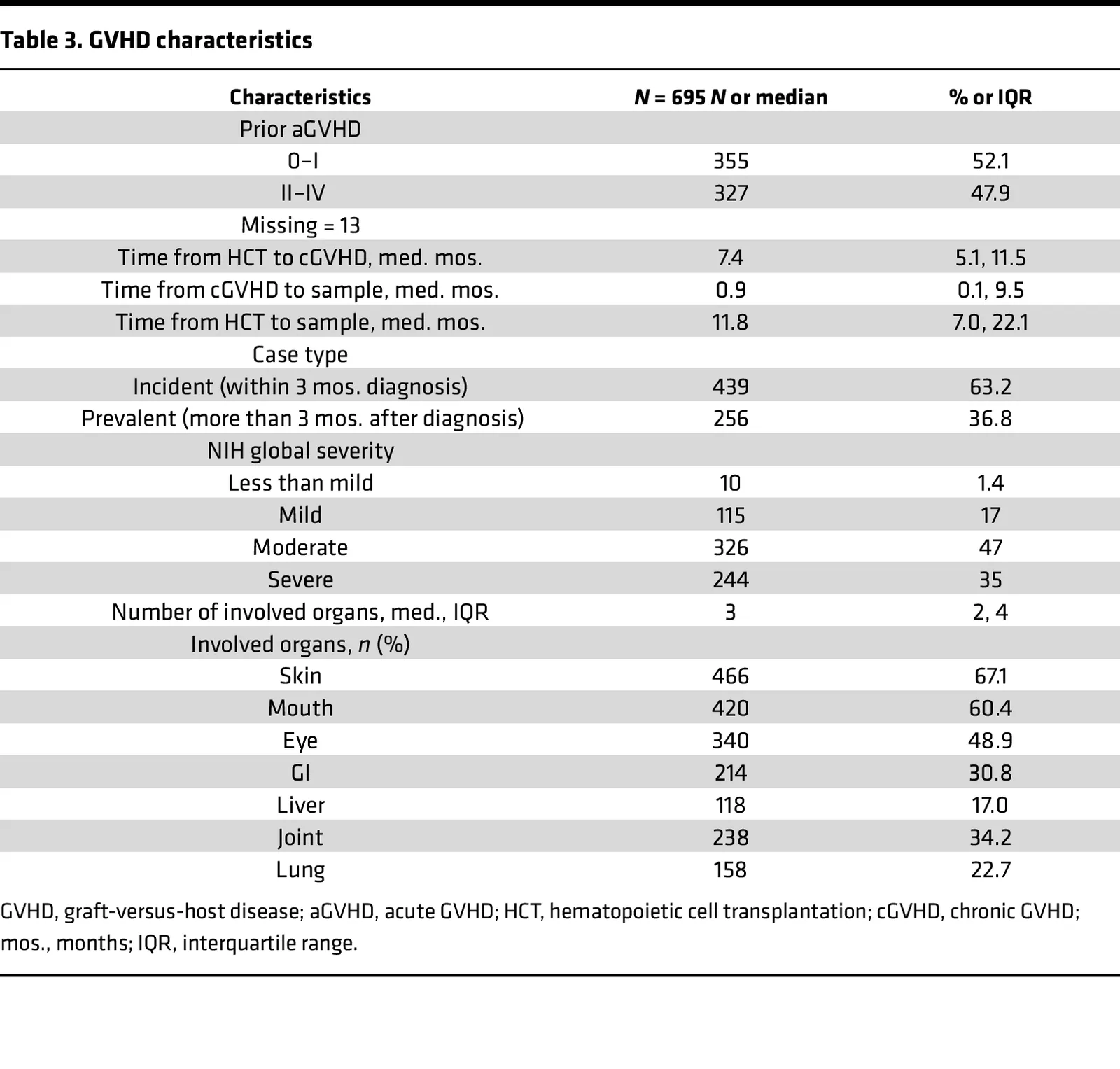
GVHD characteristics

**Table 5:**
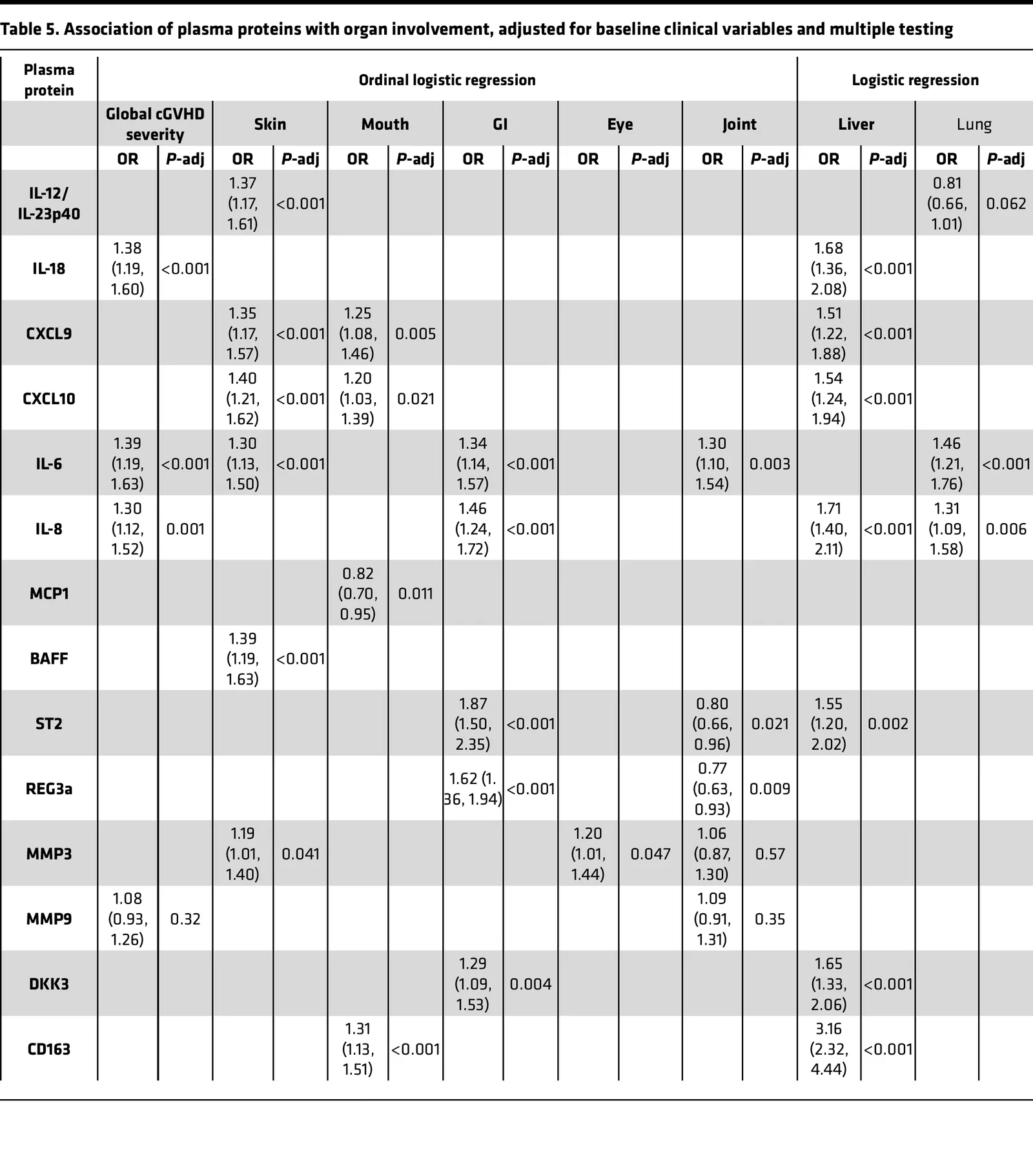
Association of plasma proteins with organ involvement, adjusted for baseline clinical variables and multiple testing

## Version 1 07/28/2026

In-Press Preview

## Version 2 09/08/2026

Electronic publication

## Version 3 09/11/2026

Corrected a technical issue related to figure display
