# Supplementary material for: Plasma proteins associated with chronic graft-versus-host disease organ involvement
Source: JCI Insight. 2026 Jul 28;11(17):e209042. doi: 10.1172/jci.insight.209042 (PMC13564087; doi:10.1172/jci.insight.209042)
Supplement: Supplementary file 1 [file jciinsight-11-209042-s008.pdf]

Supplementary Files

Plasma proteins associated with chronic graft-versus-host disease organ involvement

Stephanie J. Lee,[1] Corey Cutler,[2] Ningxin Ma,[1] Tim Randolph,[1] George L. Chen,[3] Joseph Pidala,[4] Betty K. Hamilton,[5] Carrie L. Kitko,[6] Sally Arai,[7] Najla El Jurdi,[8] Lynn Onstad,[1] Motoko Koyoma,[1] Catherine J. Lee,[1] Sophie Paczesny*,[9] Geoff R. Hill*[1]

*Co-senior authors

[1]Fred Hutchinson Cancer Center, Seattle, WA; [2]Division of Transplantation and Cellular Therapy, Dana-Farber Cancer Institute, Boston, MA; [3]The University of Texas MD Anderson Cancer Center, Houston, Tx; [4]Department of Blood and Marrow Transplantation and Cellular Immunotherapy, Moffitt Cancer Center, Tampa, FL; [5]Taussig Cancer Institute, Cleveland Clinic, Cleveland, OH; [6]Vanderbilt University Medical Center, Nashville, TN; [7]Stanford Blood and Marrow Transplantation and Cellular Therapy Division, Stanford School of Medicine, Stanford University, Stanford, CA; [8]Immune Deficiency Cellular Therapy Program, Center for Cancer Research, National Cancer Institute, National Institutes of Health, Bethesda, MD; [9]Hollings Cancer Center and Department of Pharmacology and Immunology, Medical University of South Carolina, Charleston, SC

Supplementary Table 1. Participant characteristics

	N	%
All	695	100.0
Study site		
Fred Hutchinson	357	51.4
Vanderbilt	68	9.8
Dana-Farber	62	8.9
Roswell	39	5.6
U of Minnesota	36	5.2
Moffitt	36	5.2
Cleveland Clinic	31	4.5
U of British Columbia	23	3.3
Stanford	18	2.6
Others[1]	25	3.6
Sex		
Female	264	38.0
Male	431	62.0
Age, median years, IQR	54	40, 62
Patient race		
White	612	88.1
Asian	33	4.7
Black or African American	17	2.4
American Indian/Alaska Native	6	0.9
Native Hawaiian or Other Pacific Islander	4	0.6
Multi-Race	4	0.6
Unknown	17	2.4
Other	2	0.3
Hispanic		
Not Hispanic descent	644	92.7
Hispanic descent	35	5.0
Unknown	16	2.3

	N	%
Disease diagnosis		
Acute myeloid leukemia	235	33.8
Myelodysplastic syndrome	111	16.0
Acute lymphoblastic leukemia	88	12.7
Chronic myeloid leukemia	41	5.9
Chronic lymphocytic leukemia	41	5.9
Non-Hodgkin lymphoma	74	10.7
Hodgkin lymphoma	20	2.9
Myeloproliferative neoplasm	29	4.2
Multiple myeloma	22	3.2
Aplastic anemia	9	1.3
Myelofibrosis	7	1.0
MPN	4	0.6
Other leukemia	6	0.9
Non-malignant	8	1.2
Disease status		
Early	319	45.9
Intermediate	266	38.3
Advanced	110	15.8
Transplant source		
Peripheral blood	628	90.4
Bone marrow	44	6.3
Cord blood	21	3.0
Missing	2	0.3
Donor match		
Matched unrelated donor	347	49.9
Mismatched unrelated donor	91	13.1
HLA-identical sibling	224	32.2
HLA-matched other relative	17	2.4
HLA mismatched relative	10	1.4
Haploidentical relative	5	0.7

	N	%
Missing	1	0.1
Conditioning intensity		
Myeloablative	322	46.3
Not myeloablative	372	53.5
Missing	1	0.1
Female donor, male recipient		
Female donor, male recipient	182	26.2
Other sex combination	509	73.2
Missing	4	0.6
Acute GVHD prophylaxis		
CNI+MTX	369	53.1
CNI+other (not MTX or PTCY)	304	43.7
PTCy	9	1.3
T-cell depletion	3	0.4
Other[2]	7	1.0
None	3	0.4

[1]Others: Duke (7), MD Anderson (7), Weill Cornell (4), Ohio State (2), Medical College of Wisconsin (2), Washington University (2), University of North Carolina (1)
[2]Other: MMF only (2), MTX only (3), MTX+MMF (1), MMF+other (1)

Supplementary Table 2. Graft-versus-host disease characteristics

Characteristics	N=695<br>N or median	% or IQR
Source of participants		
Study 1: NCT00637689	146	21.0
Study 2: NCT01206309	201	28.9
Study 3: NCT01902576	348	50.1
Skin severity		
None	228	33
Score 1	128	19
Score 2	170	24
Score 3	168	24
Missing=1		
Mouth severity		
None	275	40
Score 1	316	45
Score 2	90	13
Score 3	14	2
Eye severity		
None	352	51
Score 1	224	32
Score 2	95	14
Score 3	21	3
Missing=3		
Gastrointestinal severity		
None	479	69
Score 1	147	21
Score 2	59	8.5
Score 3	8	1.2
Missing=2		
Liver severity		
None	560	83
Score 1	42	6.2
Score 2	65	9.6
Score 3	11	1.6
Missing=17		
Joint severity		
None	445	65
Score 1	147	22
Score 2	79	12
Score 3	12	1.8
Missing=12		
Lung severity		
None	531	77
Score 1	117	17
Score 2	28	4.1

Score 3<br>Missing=6	13	1.9

Abbreviations: GVHD, graft-versus-host disease; HCT, hematopoietic cell transplantation; cGVHD, chronic GVHD; mos, months; IQR, interquartile range

Supplementary Table 3. Association of plasma proteins with organ involvement, adjusted for multiple testing but not adjusted for clinical variables.

Plasma protein	Ordinal Logistic Regression												Logistic Regression			
	global cGVHD severity		Skin		Mouth		Gi		Eye		Joint		Liver		Lung	
	OR	padj	OR	padj	OR	padj	OR	padj	OR	padj	OR	padj	OR	padj	OR	padj
**IL-12/IL23p40**	1.05 (0.92, 1.21)	0.65	1.21 (1.06, 1.39)	0.026	1.16 (1.01, 1.33)	0.12	0.87 (0.74, 1.02)	0.23	0.88 (0.77, 1.01)	0.21	1.02 (0.87, 1.19)	0.88	1.11 (0.91, 1.36)	0.51	0.75 (0.63, 0.90)	0.011
**IL-12p70**	1.05 (0.91, 1.21)	0.68	1.08 (0.95, 1.24)	0.42	1.16 (1.00, 1.34)	0.13	0.91 (0.77, 1.07)	0.43	1.00 (0.87, 1.15)	>0.99	1.05 (0.90, 1.23)	0.68	1.04 (0.85, 1.27)	0.82	0.98 (0.82, 1.17)	0.88
**IL-18**	1.20 (1.04, 1.38)	0.047	1.07 (0.93, 1.22)	0.55	1.11 (0.96, 1.28)	0.35	1.13 (0.96, 1.32)	0.29	0.99 (0.86, 1.15)	0.95	0.88 (0.75, 1.03)	0.26	1.63 (1.34, 2.01)	<0.001	1.08 (0.90, 1.28)	0.61
**IL-17**	1.03 (0.88, 1.21)	0.83	1.13 (0.97, 1.32)	0.27	0.88 (0.75, 1.04)	0.29	1.11 (0.93, 1.33)	0.45	0.97 (0.83, 1.14)	0.83	0.98 (0.83, 1.16)	0.88	0.87 (0.69, 1.08)	0.42	0.83 (0.68, 1.02)	0.2
**IL-21**	1.07 (0.92, 1.23)	0.58	1.08 (0.94, 1.23)	0.46	1.18 (1.02, 1.36)	0.082	0.94 (0.79, 1.10)	0.61	1.04 (0.90, 1.21)	0.72	1.10 (0.94, 1.28)	0.43	1.02 (0.84, 1.25)	0.88	0.98 (0.82, 1.17)	0.88
**CXCL9**	1.18 (1.03, 1.36)	0.077	1.26 (1.10, 1.44)	0.006	1.32 (1.15, 1.53)	0.002	0.94 (0.80, 1.11)	0.66	1.02 (0.88, 1.17)	0.88	1.14 (0.98, 1.33)	0.23	1.33 (1.10, 1.62)	0.02	0.90 (0.75, 1.08)	0.45
**CXCL10**	1.15 (1.00, 1.33)	0.13	1.29 (1.13, 1.48)	0.002	1.24 (1.08, 1.43)	0.015	0.91 (0.78, 1.07)	0.44	1.00 (0.86, 1.15)	0.97	1.11 (0.95, 1.29)	0.39	1.38 (1.13, 1.70)	0.014	0.98 (0.82, 1.18)	0.91
**GMCSF**	1.10 (0.96, 1.27)	0.37	1.09 (0.95, 1.24)	0.42	1.17 (1.02, 1.35)	0.095	0.88 (0.75, 1.04)	0.29	1.06 (0.92, 1.22)	0.64	1.10 (0.94, 1.29)	0.42	0.94 (0.77, 1.15)	0.69	1.02 (0.86, 1.22)	0.88
**MCSF**	1.14 (0.99, 1.31)	0.21	1.04 (0.90, 1.19)	0.76	1.09 (0.95, 1.26)	0.42	0.93 (0.79, 1.09)	0.59	1.01 (0.88, 1.16)	0.93	1.02 (0.87, 1.18)	0.9	1.11 (0.91, 1.34)	0.5	1.09 (0.91, 1.30)	0.52
**IL-6**	1.31 (1.13, 1.52)	0.003	1.33 (1.16, 1.53)	<0.001	0.98 (0.85, 1.14)	0.88	1.26 (1.08, 1.47)	0.019	1.01 (0.87, 1.16)	0.95	1.33 (1.14, 1.55)	0.002	1.11 (0.91, 1.34)	0.5	1.38 (1.16, 1.64)	0.003
**IL-8**	1.25 (1.08, 1.45)	0.017	1.13 (0.98, 1.29)	0.23	1.02 (0.88, 1.18)	0.88	1.46 (1.25, 1.71)	<0.001	1.14 (0.99, 1.32)	0.21	0.94 (0.80, 1.10)	0.65	1.60 (1.32, 1.94)	<0.001	1.28 (1.07, 1.52)	0.032
**MCP1**	0.97 (0.84, 1.12)	0.82	1.09 (0.95, 1.24)	0.43	0.80 (0.69, 0.93)	0.017	0.89 (0.75, 1.05)	0.34	1.18 (1.02, 1.37)	0.095	1.23 (1.05, 1.45)	0.052	0.90 (0.73, 1.10)	0.49	1.24 (1.03, 1.50)	0.083
**BAFF**	1.19 (1.03, 1.37)	0.063	1.23 (1.07, 1.41)	0.018	1.04 (0.90, 1.19)	0.75	0.91 (0.77, 1.07)	0.45	0.94 (0.81, 1.09)	0.61	1.03 (0.88, 1.21)	0.81	1.19 (0.97, 1.45)	0.23	0.94 (0.79, 1.13)	0.67
**ST2**	1.02 (0.89, 1.17)	0.88	0.94 (0.82, 1.08)	0.58	1.07 (0.92, 1.24)	0.59	1.76 (1.46, 2.14)	<0.001	1.04 (0.90, 1.21)	0.74	0.78 (0.66, 0.91)	0.014	1.63 (1.30, 2.06)	<0.001	1.03 (0.87, 1.24)	0.83
**Reg3a**	1.05 (0.91, 1.21)	0.66	1.00 (0.87, 1.13)	0.96	1.18 (1.02, 1.36)	0.085	1.65 (1.39, 1.95)	<0.001	1.05 (0.91, 1.21)	0.68	0.70 (0.59, 0.82)	<0.001	1.14 (0.93, 1.38)	0.39	0.85 (0.70, 1.02)	0.22
**MMP3**	1.18 (1.02, 1.36)	0.077	1.29 (1.13, 1.48)	0.002	0.95 (0.82, 1.09)	0.65	1.14 (0.97, 1.34)	0.28	1.30 (1.13, 1.51)	0.004	1.24 (1.06, 1.45)	0.038	1.05 (0.86, 1.29)	0.75	1.10 (0.92, 1.32)	0.51
**MMP9**	1.22 (1.05, 1.40)	0.033	1.18 (1.03, 1.36)	0.057	1.11 (0.97, 1.29)	0.29	1.09 (0.93, 1.29)	0.46	1.13 (0.98, 1.30)	0.25	1.33 (1.13, 1.57)	0.005	0.78 (0.64, 0.95)	0.053	1.26 (1.05, 1.52)	0.053
**DKK3**	1.04 (0.90, 1.19)	0.76	1.14 (1.00, 1.30)	0.15	1.21 (1.04, 1.40)	0.048	1.30 (1.11, 1.53)	0.008	1.10 (0.95, 1.27)	0.39	0.94 (0.81, 1.10)	0.65	1.40 (1.15, 1.70)	0.006	0.99 (0.83, 1.19)	0.96
**CD163**	1.16 (1.01, 1.33)	0.13	1.09 (0.95, 1.24)	0.42	1.33 (1.15, 1.54)	0.001	1.04 (0.89, 1.22)	0.75	1.05 (0.91, 1.22)	0.65	0.95 (0.82, 1.11)	0.67	3.01 (2.25, 4.14)	<0.001	0.98 (0.82, 1.17)	0.88

Supplementary Table 4. Important clinical and plasma protein variables associated with individual organ involvement. Results from elastic net models over 100 runs. Shown are variables that were selected > 50 times.

organ	feature	n	coef
**Skin**	type_incident	100	-0.5
	MMP3	100	0.21
	CXCL9	100	0.17
	IL-6	100	0.16
	yrs_tx2draw	100	0.08
	age	100	0.01
	IL-12/IL23p40	94	0.11
	DKK3	92	0.07
	MCSF	58	-0.07
**Mouth**	CXCL9	77	0.09
	CD163	74	0.07
	type_incident	71	0.16
	DKK3	66	0.09
	MCP1	63	-0.07
**GI**	female	100	0.36
	ST2	100	0.32
	Reg3a	100	0.24
	IL-6	99	0.16
	IL-8	92	0.09
	CXCL10	87	-0.1
	MCP1	75	-0.06
**Eye**	type_incident	99	-0.32
	age	96	0.01
	MCP1	94	0.12
	MMP3	92	0.08
	pse_mg_d	84	0.04
	female	83	0.18
	yrs_tx2draw	69	0.04
	CD163	57	0.06
	IL-8	55	0.05
**Joint**	type_incident	100	-1.38
	IL-6	100	0.21
	yrs_tx2draw	100	0.21
	Reg3a	100	-0.19
	CXCL9	99	0.19
	ST2	98	-0.1
	MMP3	79	0.1
	MMP9	67	0.06
	IL-8	64	-0.1
**Liver**	CD163	100	0.77
	type_incident	100	0.51
	IL-8	100	0.3
	DKK3	100	0.26
	MMP9	100	-0.2
	ST2	100	0.17
	pse_mg_d	100	0.13
	yrs_tx2draw	100	-0.09
	age	100	-0.03
	IL-18	91	0.07
	MCP1	88	-0.09
	CXCL9	77	0.11
	MCSF	70	0.08
**Lung**	type_incident	100	-0.85
	female	100	0.63
	IL-6	100	0.34
	IL-12/IL23p40	100	-0.31
	Reg3a	99	-0.18
	pse_mg_d	98	0.09
	MMP9	95	0.12
	age	95	0.01
	IL-18	89	0.09
	IL-8	86	0.08
	MCP1	83	0.09
	DKK3	68	-0.06

Supplementary Table 5. Antibody sources and quality control notes

Protein	Capture Antibody	Detection Antibody	Luminex bead	Notes
Th1				
IFNa	Mabtech # 3425-3-250	Mabtech # 3425-6-250	MC10020-01	Failed quality control – values too high in normal controls
IFNg	Fisher Scientific # M700A	Fisher Scientific # M700A	MC10033-01	Failed quality control – values too high in normal controls
TNFa	BD CBA Set # 560112 (Capture bead # 51-9005468)	BD CBA Set # 560112 (Detection reagent # 51-9004040)		Levels below detectable
IL-2	BD CBA Set # 558270 (Capture bead # 51-9005283)	BD CBA Set # 558270 (Detection reagent # 51-9004034		Levels below detectable
IL-1b	BD CBA Set # 558279 (Capture bead # 51-9005302)	BD CBA Set # 558279 (Detection reagent # 51-9004060)		Levels below detectable
**IL-12**	BD Biosciences # 555065	BD Biosciences # 554660	MC10051-01	
**IL-12/IL-23p40**	BD CBA Set # 560154 (Capture bead # 51-9005602)	BD CBA Set # 560154 (Detection reagent # 51-9005600)		
**IL-18**	R&D Systems # D044-3	R&D Systems # D045-6	MC10008-01	
**CXCL9 (MIG)**	R&D Systems # MAB392	R&D Systems # BAF392	MC10088-01	
**CXCL10 (IP10)**	R&D Systems # MAB266	R&D Systems # BAF266	MC10073-01	
Th2				
IL-4	BD CBA Set # 558272 (Capture bead # 51-9005295)	BD CBA Set # 558272 (Detection reagent # 51-9004037)		Levels below detectable
IL-10	BD CBA Set # 558274 (Capture bead # 51-9005291)	BD CBA Set # 558274 (Detection reagent # 51-9004043)		Levels below detectable
IL-13	BD CBA Set # 558450 (Capture bead # 51-9005346)	BD CBA Set # 558450 (Detection reagent # 51-9004624)		Levels below detectable
Th17/Tc17				
IL-17a	BD CBA set # 560383 (Capture bead # 51-9006383)	BD CBA set # 560383 (Detection reagent # 51-9006081)		Levels below detectable
**IL-17**	R&D Quantikine® HS	R&D Systems | Catalog # HS170		Precoated capture antibody, pairs not disclosed by the company
**IL-21**	MC10054-01	Mabtech # 3540-6-250	MC10054-01	

IL-23				Levels below detectable
Monocyte				
**GM-CSF**	BD Biosciences # 554502	BD Biosciences # 554505	MC10045-01	
**M-CSF**	R&D Systems # MAB616	R&D Systems # BAF216	MC10077-01	
**IL-6**	BD CBA Set # 558276 (Capture bead # 51-9005287)	BD CBA Set # 558276 (Detection reagent # 51-9004046)		
**IL-8**	BD CBA Set # 558277 (Capture bead # 51-9005289)	BD CBA Set # 558277 (Detection reagent # 51-9004049)		
**MCP-1**	BD CBA Set # 558287 (Capture bead # 51-9005310)	BD CBA Set # 558287 (Detection reagent # 51-9004072)		
B cells				
**BAFF**	R&D Systems # AF124	Sino Biological # 10056-RP02	MC10065-01	
Others				
**ST2/IL33R**	R&D Quantikine	R&D Systems | Catalog # DST200		Precoated capture antibody
**Reg3a**	R&D Systems # AF5940	Sino Biological # 11235-R019	MC10089-01	
**MMP3**	R&D Duoset	R&D Systems | Catalog # DY513		
**MMP9**	R&D Duoset	R&D Systems | Catalog # DY911		
**DKK3**	R&D Duoset	R&D Systems | Catalog # DY1118		
**CD163**	R&D Quantikine	R&D Systems | Catalog # DC1630		Precoated capture antibody

Supplementary Figures

Supplementary Figure 1. Correlation of plasma proteins. IL-17 correlation calculated on 544 patients instead of 695

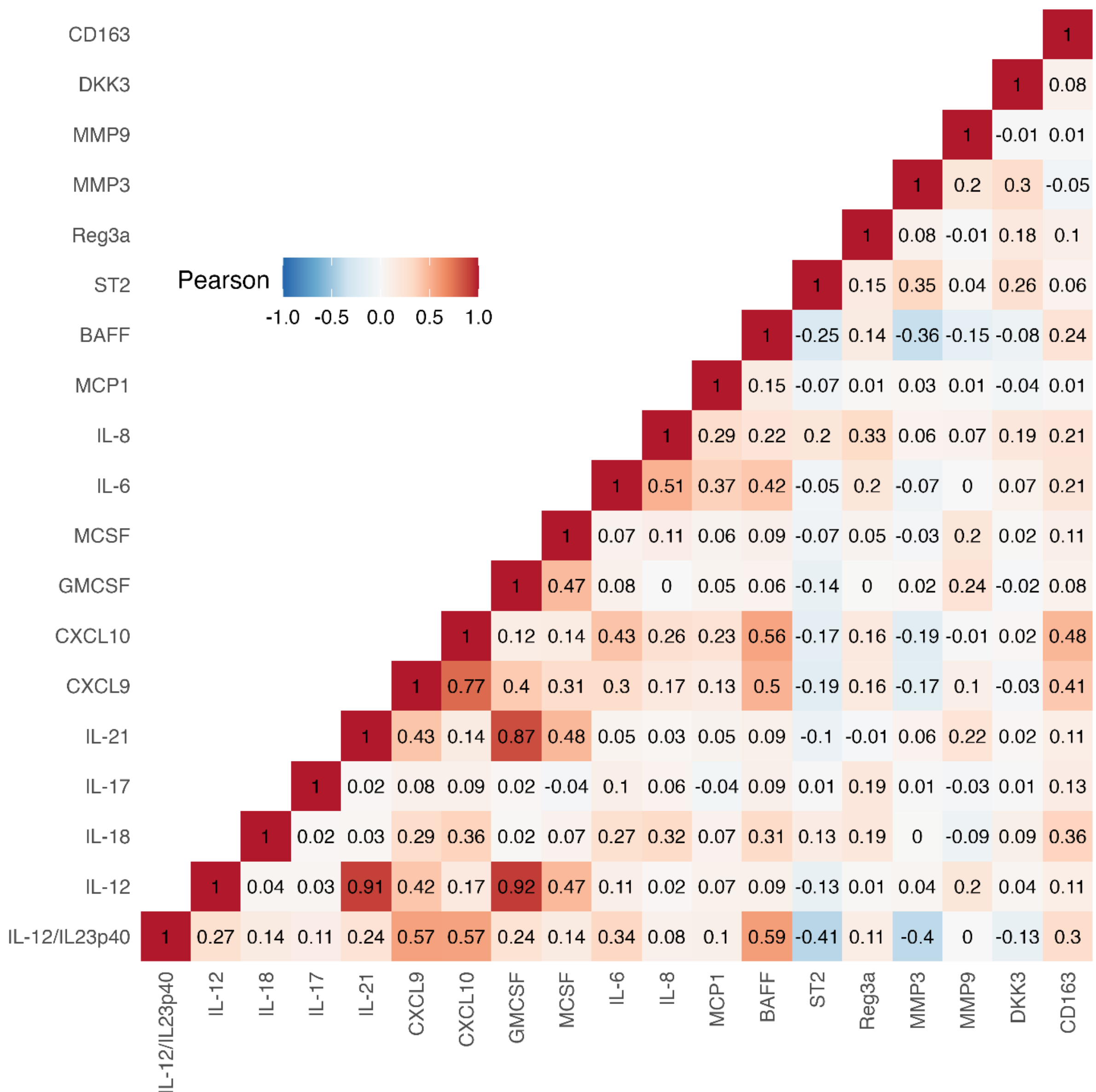
Pearson
-1.0 -0.5 0.0 0.5 1.0
CD163 1
DKK3 1 0.08
MMP9 1 -0.01 0.01
MMP3 1 0.2 0.3 -0.05
Reg3a 1 0.08 -0.01 0.18 0.1
ST2 1 0.15 0.35 0.04 0.26 0.06
BAFF 1 -0.25 0.14 -0.36 -0.15 -0.08 0.24
MCP1 1 0.15 -0.07 0.01 0.03 0.01 -0.04 0.01
IL-8 1 0.29 0.22 0.2 0.33 0.06 0.07 0.19 0.21
IL-6 1 0.51 0.37 0.42 -0.05 0.2 -0.07 0 0.07 0.21
MCSF 1 0.07 0.11 0.06 0.09 -0.07 0.05 -0.03 0.2 0.02 0.11
GMCSF 1 0.47 0.08 0 0.05 0.06 -0.14 0 0.02 0.24 -0.02 0.08
CXCL10 1 0.12 0.14 0.43 0.26 0.23 0.56 -0.17 0.16 -0.19 -0.01 0.02 0.48
CXCL9 1 0.77 0.4 0.31 0.3 0.17 0.13 0.5 -0.19 0.16 -0.17 0.1 -0.03 0.41
IL-21 1 0.43 0.14 0.87 0.48 0.05 0.03 0.05 0.09 -0.1 -0.01 0.06 0.22 0.02 0.11
IL-17 1 0.02 0.08 0.09 0.02 -0.04 0.1 0.06 -0.04 0.09 0.01 0.19 0.01 -0.03 0.01 0.13
IL-18 1 0.02 0.03 0.29 0.36 0.02 0.07 0.27 0.32 0.07 0.31 0.13 0.19 0 -0.09 0.09 0.36
IL-12 1 0.04 0.03 0.91 0.42 0.17 0.92 0.47 0.11 0.02 0.07 0.09 -0.13 0.01 0.04 0.2 0.04 0.11
IL-12/IL23p40 1 0.27 0.14 0.11 0.24 0.57 0.57 0.24 0.14 0.34 0.08 0.1 0.59 -0.41 0.11 -0.4 0 -0.13 0.3
IL-12/IL23p40
IL-12
IL-18
IL-17
IL-21
CXCL9
CXCL10
GMCSF
MCSF
IL-6
IL-8
MCP1
BAFF
ST2
Reg3a
MMP3
MMP9
DKK3
CD163

Supplementary Figure 2. Panel A. Averaged receiver operating characteristics curves for logistic regression models predicting joint involvement using different predictor sets. Curves represent the mean test ROC across 100 random train-test splits. Shaded areas indicate the empirical 95% interval of sensitivity. **Panel B.** KRV coefficients of plasma protein profiles and individual organ involvement.

Panel A

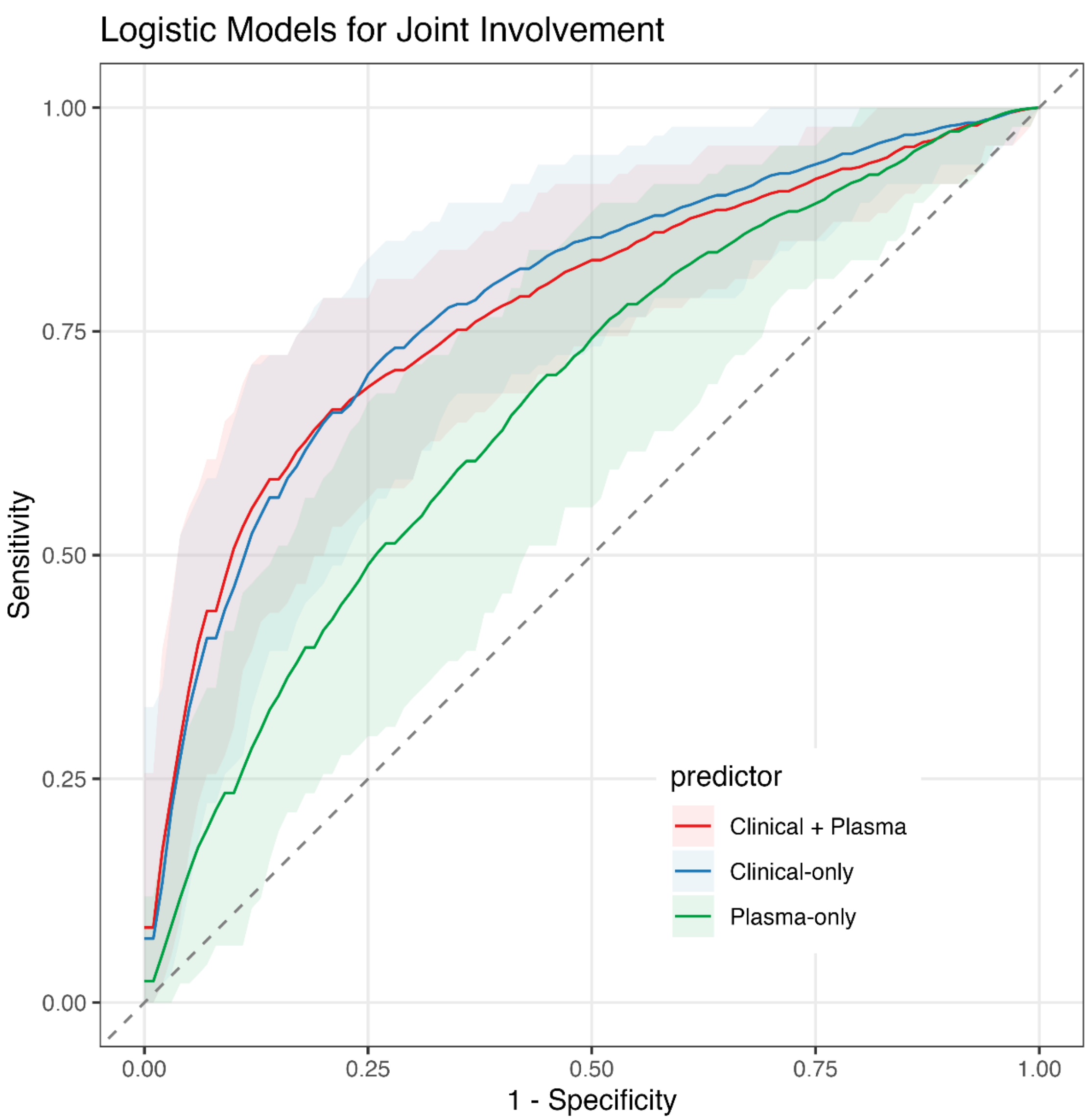

Averaged AUC of different predictor sets on joint involvement

	Clinical + Plasma Protein	Plasma Protein
Logistic*	0.77 (0.70, 0.84)	0.67 (0.57, 0.75)
Elastic net	0.79 (0.72, 0.86)	0.67 (0.58, 0.74)
Random Forest	0.81 (0.74, 0.88)	0.64 (0.56, 0.70)
XGBoost	0.80 (0.75, 0.87)	0.63 (0.56, 0.70)
BART	0.80 (0.72, 0.86)	0.65 (0.58, 0.73)

*AUC of the logistic model using only clinical variables is 0.78 (0.71, 0.85).

Panel B

Outcomes	p-value	R^2
Skin	2.7e-05	0.05%
Mouth	0.0005	0.03%
Eye	0.05	0.01%
Gastrointestinal	6.5e-07	0.08%
Liver	8.5e-09	0.1%
Joint	8.6e-05	0.04%
Lung	0.008	0.02%
All 7 single organ cGVHD scores	4.4e-16	0.3%
NIH global score	0.0037	0.02%
